# Supplementary material for: Modeling glioblastoma heterogeneity as a dynamic network of cell states
Source: Mol Syst Biol. 2021 Sep 16;17(9):e10105. doi: 10.15252/msb.202010105 (PMC8444284; doi:10.15252/msb.202010105)
Supplement: Supplementary file 5 — Source Data for Figure 3 [file MSB-17-e10105-s001.zip › Figure3A_sourcedata/GSEA_3065/nosigdb_state2.GseaPreranked.1622718419215/RICHARDS_NOWAKOWSKI_SCIENCE_2017_RG_EARLY_UPREG.html]

Details for gene set RICHARDS\_NOWAKOWSKI\_SCIENCE\_2017\_RG\_EARLY\_UPREG[GSEA]

|  || Dataset | state2 |
| Phenotype | NoPhenotypeAvailable |
| Upregulated in class | na\_pos |
| GeneSet | RICHARDS\_NOWAKOWSKI\_SCIENCE\_2017\_RG\_EARLY\_UPREG |
| Enrichment Score (ES) | 0.7151237 |
| Normalized Enrichment Score (NES) | 2.752885 |
| Nominal p-value | 0.0 |
| FDR q-value | 0.0 |
| FWER p-Value | 0.0 |
Table: GSEA Results Summary

  

Fig 1: Enrichment plot: RICHARDS\_NOWAKOWSKI\_SCIENCE\_2017\_RG\_EARLY\_UPREG      
 Profile of the Running ES Score & Positions of GeneSet Members on the Rank Ordered List

  

| PROBE | GENE SYMBOL | GENE\_TITLE | RANK IN GENE LIST | RANK METRIC SCORE | RUNNING ES | CORE ENRICHMENT || 1 | HIST1H4C |  |  | 0 | 1.309 | 0.0176 | Yes |
| 2 | HIST1H1B |  |  | 1 | 0.902 | 0.0297 | Yes |
| 3 | HIST1H1D |  |  | 3 | 0.842 | 0.0409 | Yes |
| 4 | HMGB2 |  |  | 4 | 0.823 | 0.0520 | Yes |
| 5 | TYMS |  |  | 5 | 0.792 | 0.0626 | Yes |
| 6 | CENPU |  |  | 6 | 0.763 | 0.0729 | Yes |
| 7 | HIST1H1A |  |  | 7 | 0.760 | 0.0831 | Yes |
| 8 | H2AFZ |  |  | 8 | 0.735 | 0.0929 | Yes |
| 9 | UBE2C |  |  | 9 | 0.704 | 0.1024 | Yes |
| 10 | UBE2T |  |  | 12 | 0.643 | 0.1108 | Yes |
| 11 | TOP2A |  |  | 15 | 0.596 | 0.1186 | Yes |
| 12 | PCNA |  |  | 16 | 0.595 | 0.1266 | Yes |
| 13 | MKI67 |  |  | 17 | 0.590 | 0.1345 | Yes |
| 14 | CDK1 |  |  | 18 | 0.577 | 0.1422 | Yes |
| 15 | MAD2L1 |  |  | 20 | 0.563 | 0.1497 | Yes |
| 16 | ATAD2 |  |  | 21 | 0.563 | 0.1573 | Yes |
| 17 | RRM2 |  |  | 22 | 0.556 | 0.1647 | Yes |
| 18 | CKS1B |  |  | 23 | 0.554 | 0.1722 | Yes |
| 19 | DUT |  |  | 24 | 0.552 | 0.1796 | Yes |
| 20 | HMGB1 |  |  | 25 | 0.549 | 0.1870 | Yes |
| 21 | CKS2 |  |  | 28 | 0.532 | 0.1939 | Yes |
| 22 | TPX2 |  |  | 29 | 0.531 | 0.2010 | Yes |
| 23 | DEK |  |  | 31 | 0.526 | 0.2080 | Yes |
| 24 | CENPF |  |  | 34 | 0.518 | 0.2147 | Yes |
| 25 | HMGN2 |  |  | 35 | 0.512 | 0.2216 | Yes |
| 26 | HIST1H1C |  |  | 38 | 0.498 | 0.2281 | Yes |
| 27 | SMC2 |  |  | 39 | 0.493 | 0.2347 | Yes |
| 28 | DBF4 |  |  | 41 | 0.492 | 0.2412 | Yes |
| 29 | SMC4 |  |  | 42 | 0.489 | 0.2478 | Yes |
| 30 | CENPK |  |  | 44 | 0.488 | 0.2542 | Yes |
| 31 | ZWINT |  |  | 45 | 0.488 | 0.2608 | Yes |
| 32 | PBK |  |  | 46 | 0.484 | 0.2673 | Yes |
| 33 | NUSAP1 |  |  | 47 | 0.483 | 0.2737 | Yes |
| 34 | MCM3 |  |  | 51 | 0.464 | 0.2796 | Yes |
| 35 | UHRF1 |  |  | 57 | 0.454 | 0.2852 | Yes |
| 36 | NCAPG |  |  | 60 | 0.445 | 0.2909 | Yes |
| 37 | BIRC5 |  |  | 61 | 0.443 | 0.2969 | Yes |
| 38 | SIVA1 |  |  | 62 | 0.439 | 0.3028 | Yes |
| 39 | PTTG1 |  |  | 63 | 0.439 | 0.3087 | Yes |
| 40 | GMNN |  |  | 64 | 0.435 | 0.3145 | Yes |
| 41 | HIST1H1E |  |  | 66 | 0.429 | 0.3202 | Yes |
| 42 | CCNB1 |  |  | 68 | 0.420 | 0.3257 | Yes |
| 43 | EZH2 |  |  | 70 | 0.417 | 0.3312 | Yes |
| 44 | LSM4 |  |  | 73 | 0.410 | 0.3365 | Yes |
| 45 | SNRPD1 |  |  | 74 | 0.408 | 0.3420 | Yes |
| 46 | MCM4 |  |  | 79 | 0.399 | 0.3469 | Yes |
| 47 | KIF4A |  |  | 84 | 0.393 | 0.3517 | Yes |
| 48 | KIF15 |  |  | 86 | 0.392 | 0.3569 | Yes |
| 49 | CENPN |  |  | 87 | 0.390 | 0.3621 | Yes |
| 50 | RAD51AP1 |  |  | 90 | 0.388 | 0.3671 | Yes |
| 51 | BRCA1 |  |  | 92 | 0.381 | 0.3721 | Yes |
| 52 | MND1 |  |  | 96 | 0.376 | 0.3768 | Yes |
| 53 | CDKN3 |  |  | 97 | 0.376 | 0.3818 | Yes |
| 54 | RAN |  |  | 98 | 0.375 | 0.3869 | Yes |
| 55 | TMSB15A |  |  | 99 | 0.374 | 0.3919 | Yes |
| 56 | SPC25 |  |  | 104 | 0.372 | 0.3965 | Yes |
| 57 | CENPE |  |  | 105 | 0.372 | 0.4015 | Yes |
| 58 | CACYBP |  |  | 109 | 0.366 | 0.4060 | Yes |
| 59 | RHEB |  |  | 110 | 0.362 | 0.4109 | Yes |
| 60 | ANLN |  |  | 111 | 0.358 | 0.4157 | Yes |
| 61 | NDC80 |  |  | 115 | 0.354 | 0.4201 | Yes |
| 62 | KIF23 |  |  | 117 | 0.351 | 0.4247 | Yes |
| 63 | ASPM |  |  | 122 | 0.342 | 0.4289 | Yes |
| 64 | PTGES3 |  |  | 128 | 0.336 | 0.4328 | Yes |
| 65 | KIF11 |  |  | 129 | 0.335 | 0.4373 | Yes |
| 66 | SNRPB |  |  | 132 | 0.332 | 0.4416 | Yes |
| 67 | CCNA2 |  |  | 135 | 0.331 | 0.4458 | Yes |
| 68 | ANP32E |  |  | 136 | 0.330 | 0.4502 | Yes |
| 69 | USP1 |  |  | 138 | 0.329 | 0.4546 | Yes |
| 70 | SRSF3 |  |  | 140 | 0.327 | 0.4588 | Yes |
| 71 | RBBP8 |  |  | 141 | 0.325 | 0.4632 | Yes |
| 72 | SNRPG |  |  | 144 | 0.322 | 0.4673 | Yes |
| 73 | ARHGAP11A |  |  | 145 | 0.321 | 0.4716 | Yes |
| 74 | CKAP2 |  |  | 151 | 0.308 | 0.4752 | Yes |
| 75 | KIF22 |  |  | 154 | 0.306 | 0.4791 | Yes |
| 76 | SNRPE |  |  | 157 | 0.306 | 0.4830 | Yes |
| 77 | CDCA3 |  |  | 160 | 0.304 | 0.4868 | Yes |
| 78 | HELLS |  |  | 161 | 0.304 | 0.4909 | Yes |
| 79 | ECT2 |  |  | 171 | 0.297 | 0.4939 | Yes |
| 80 | BTG3 |  |  | 173 | 0.295 | 0.4978 | Yes |
| 81 | PSIP1 |  |  | 175 | 0.294 | 0.5016 | Yes |
| 82 | HSPD1 |  |  | 176 | 0.294 | 0.5056 | Yes |
| 83 | ITGB3BP |  |  | 177 | 0.294 | 0.5095 | Yes |
| 84 | DDX39A |  |  | 183 | 0.293 | 0.5129 | Yes |
| 85 | BARD1 |  |  | 185 | 0.292 | 0.5167 | Yes |
| 86 | PPIA |  |  | 189 | 0.287 | 0.5202 | Yes |
| 87 | CCNB2 |  |  | 190 | 0.286 | 0.5241 | Yes |
| 88 | HMGB3 |  |  | 200 | 0.282 | 0.5269 | Yes |
| 89 | NCAPG2 |  |  | 201 | 0.282 | 0.5307 | Yes |
| 90 | CYCS |  |  | 207 | 0.279 | 0.5338 | Yes |
| 91 | KIF20B |  |  | 208 | 0.278 | 0.5376 | Yes |
| 92 | HSPE1 |  |  | 211 | 0.276 | 0.5411 | Yes |
| 93 | H1FX |  |  | 212 | 0.276 | 0.5448 | Yes |
| 94 | CHEK1 |  |  | 224 | 0.266 | 0.5471 | Yes |
| 95 | PPP1CC |  |  | 246 | 0.253 | 0.5482 | Yes |
| 96 | KIF2C |  |  | 252 | 0.251 | 0.5510 | Yes |
| 97 | SPC24 |  |  | 256 | 0.248 | 0.5540 | Yes |
| 98 | MIS18BP1 |  |  | 257 | 0.246 | 0.5573 | Yes |
| 99 | TIMELESS |  |  | 272 | 0.243 | 0.5590 | Yes |
| 100 | HAUS8 |  |  | 280 | 0.242 | 0.5615 | Yes |
| 101 | PRR11 |  |  | 285 | 0.238 | 0.5642 | Yes |
| 102 | TPI1 |  |  | 301 | 0.231 | 0.5657 | Yes |
| 103 | KIF14 |  |  | 302 | 0.231 | 0.5688 | Yes |
| 104 | GSTP1 |  |  | 306 | 0.230 | 0.5715 | Yes |
| 105 | RPL41 |  |  | 313 | 0.227 | 0.5739 | Yes |
| 106 | CKAP2L |  |  | 327 | 0.224 | 0.5755 | Yes |
| 107 | TMEM97 |  |  | 331 | 0.223 | 0.5781 | Yes |
| 108 | RACGAP1 |  |  | 333 | 0.222 | 0.5810 | Yes |
| 109 | NDUFA6 |  |  | 338 | 0.222 | 0.5836 | Yes |
| 110 | LDHB |  |  | 343 | 0.219 | 0.5861 | Yes |
| 111 | NPM1 |  |  | 351 | 0.216 | 0.5882 | Yes |
| 112 | SNRPA1 |  |  | 361 | 0.214 | 0.5900 | Yes |
| 113 | GLO1 |  |  | 363 | 0.214 | 0.5928 | Yes |
| 114 | RPS2 |  |  | 368 | 0.212 | 0.5952 | Yes |
| 115 | DLGAP5 |  |  | 371 | 0.211 | 0.5978 | Yes |
| 116 | CENPW |  |  | 373 | 0.211 | 0.6005 | Yes |
| 117 | NAP1L1 |  |  | 387 | 0.207 | 0.6019 | Yes |
| 118 | HMGN3 |  |  | 388 | 0.207 | 0.6047 | Yes |
| 119 | GNG5 |  |  | 389 | 0.207 | 0.6074 | Yes |
| 120 | RBM8A |  |  | 403 | 0.199 | 0.6087 | Yes |
| 121 | CCT4 |  |  | 405 | 0.199 | 0.6112 | Yes |
| 122 | RPL23A |  |  | 406 | 0.199 | 0.6139 | Yes |
| 123 | RNPS1 |  |  | 410 | 0.198 | 0.6162 | Yes |
| 124 | RPS25 |  |  | 413 | 0.197 | 0.6186 | Yes |
| 125 | RPL35 |  |  | 415 | 0.197 | 0.6212 | Yes |
| 126 | POLR2J |  |  | 417 | 0.196 | 0.6237 | Yes |
| 127 | HINT1 |  |  | 418 | 0.196 | 0.6263 | Yes |
| 128 | KIF18A |  |  | 419 | 0.195 | 0.6290 | Yes |
| 129 | GPX4 |  |  | 425 | 0.194 | 0.6310 | Yes |
| 130 | SPAG5 |  |  | 439 | 0.192 | 0.6321 | Yes |
| 131 | FANCD2 |  |  | 447 | 0.191 | 0.6339 | Yes |
| 132 | RPLP0 |  |  | 451 | 0.190 | 0.6361 | Yes |
| 133 | RTKN2 |  |  | 463 | 0.188 | 0.6374 | Yes |
| 134 | SSB |  |  | 478 | 0.184 | 0.6384 | Yes |
| 135 | KNSTRN |  |  | 483 | 0.183 | 0.6404 | Yes |
| 136 | PGAM1 |  |  | 487 | 0.183 | 0.6425 | Yes |
| 137 | TCP1 |  |  | 490 | 0.182 | 0.6447 | Yes |
| 138 | RPS15 |  |  | 492 | 0.182 | 0.6471 | Yes |
| 139 | PSMB3 |  |  | 502 | 0.180 | 0.6485 | Yes |
| 140 | LDHA |  |  | 506 | 0.179 | 0.6505 | Yes |
| 141 | YWHAE |  |  | 511 | 0.178 | 0.6525 | Yes |
| 142 | PRC1 |  |  | 514 | 0.178 | 0.6547 | Yes |
| 143 | RPL7 |  |  | 518 | 0.178 | 0.6567 | Yes |
| 144 | RPL18A |  |  | 523 | 0.177 | 0.6587 | Yes |
| 145 | RPS23 |  |  | 530 | 0.176 | 0.6604 | Yes |
| 146 | CTNNAL1 |  |  | 532 | 0.176 | 0.6626 | Yes |
| 147 | RPS26 |  |  | 550 | 0.172 | 0.6630 | Yes |
| 148 | RPL8 |  |  | 553 | 0.172 | 0.6651 | Yes |
| 149 | BUB1B |  |  | 572 | 0.168 | 0.6654 | Yes |
| 150 | RPS8 |  |  | 584 | 0.166 | 0.6664 | Yes |
| 151 | UQCRFS1 |  |  | 585 | 0.165 | 0.6686 | Yes |
| 152 | RPS3 |  |  | 598 | 0.164 | 0.6695 | Yes |
| 153 | HSP90AA1 |  |  | 600 | 0.164 | 0.6716 | Yes |
| 154 | KIF5B |  |  | 604 | 0.163 | 0.6734 | Yes |
| 155 | TOMM5 |  |  | 610 | 0.162 | 0.6750 | Yes |
| 156 | RPL12 |  |  | 611 | 0.162 | 0.6772 | Yes |
| 157 | CKB |  |  | 647 | 0.157 | 0.6754 | Yes |
| 158 | RPLP1 |  |  | 648 | 0.156 | 0.6775 | Yes |
| 159 | RPL32 |  |  | 661 | 0.155 | 0.6783 | Yes |
| 160 | RPL29 |  |  | 673 | 0.153 | 0.6791 | Yes |
| 161 | NUP50 |  |  | 685 | 0.152 | 0.6799 | Yes |
| 162 | NOP58 |  |  | 689 | 0.151 | 0.6816 | Yes |
| 163 | RPS24 |  |  | 703 | 0.150 | 0.6822 | Yes |
| 164 | ID4 |  |  | 705 | 0.150 | 0.6841 | Yes |
| 165 | PUF60 |  |  | 712 | 0.149 | 0.6854 | Yes |
| 166 | ENO1 |  |  | 717 | 0.148 | 0.6870 | Yes |
| 167 | RPS3A |  |  | 719 | 0.148 | 0.6889 | Yes |
| 168 | NOL7 |  |  | 728 | 0.146 | 0.6899 | Yes |
| 169 | NFYB |  |  | 740 | 0.145 | 0.6907 | Yes |
| 170 | EEF1D |  |  | 783 | 0.140 | 0.6879 | Yes |
| 171 | CSNK2B |  |  | 786 | 0.140 | 0.6895 | Yes |
| 172 | CEP135 |  |  | 795 | 0.138 | 0.6905 | Yes |
| 173 | RPS6 |  |  | 819 | 0.136 | 0.6898 | Yes |
| 174 | H3F3B |  |  | 829 | 0.135 | 0.6906 | Yes |
| 175 | TTK |  |  | 831 | 0.135 | 0.6923 | Yes |
| 176 | RPS14 |  |  | 834 | 0.134 | 0.6939 | Yes |
| 177 | RPS7 |  |  | 849 | 0.133 | 0.6941 | Yes |
| 178 | SYNE2 |  |  | 853 | 0.132 | 0.6955 | Yes |
| 179 | DPY30 |  |  | 854 | 0.132 | 0.6973 | Yes |
| 180 | RPL19 |  |  | 862 | 0.131 | 0.6983 | Yes |
| 181 | PSMA3 |  |  | 866 | 0.131 | 0.6997 | Yes |
| 182 | MRPL47 |  |  | 891 | 0.129 | 0.6988 | Yes |
| 183 | UBB |  |  | 930 | 0.125 | 0.6963 | Yes |
| 184 | GAPDH |  |  | 933 | 0.125 | 0.6977 | Yes |
| 185 | RPL15 |  |  | 936 | 0.125 | 0.6992 | Yes |
| 186 | PTBP1 |  |  | 943 | 0.124 | 0.7002 | Yes |
| 187 | RPL13 |  |  | 958 | 0.123 | 0.7003 | Yes |
| 188 | RPL6 |  |  | 968 | 0.122 | 0.7009 | Yes |
| 189 | RPL10A |  |  | 984 | 0.121 | 0.7009 | Yes |
| 190 | RPS12 |  |  | 985 | 0.121 | 0.7025 | Yes |
| 191 | PSMB7 |  |  | 989 | 0.121 | 0.7038 | Yes |
| 192 | MED30 |  |  | 992 | 0.120 | 0.7052 | Yes |
| 193 | RPL30 |  |  | 1018 | 0.118 | 0.7040 | Yes |
| 194 | TWISTNB |  |  | 1019 | 0.118 | 0.7056 | Yes |
| 195 | SCAND1 |  |  | 1024 | 0.118 | 0.7067 | Yes |
| 196 | SOX2 |  |  | 1036 | 0.117 | 0.7071 | Yes |
| 197 | CEP152 |  |  | 1047 | 0.116 | 0.7075 | Yes |
| 198 | PPA1 |  |  | 1052 | 0.115 | 0.7086 | Yes |
| 199 | RPL14 |  |  | 1057 | 0.115 | 0.7097 | Yes |
| 200 | RPS27A |  |  | 1072 | 0.113 | 0.7097 | Yes |
| 201 | RPL7A |  |  | 1073 | 0.113 | 0.7112 | Yes |
| 202 | RPL39 |  |  | 1074 | 0.113 | 0.7127 | Yes |
| 203 | TCF3 |  |  | 1077 | 0.113 | 0.7140 | Yes |
| 204 | RPLP2 |  |  | 1099 | 0.112 | 0.7132 | Yes |
| 205 | RPL22L1 |  |  | 1112 | 0.111 | 0.7133 | Yes |
| 206 | RPL21 |  |  | 1123 | 0.110 | 0.7137 | Yes |
| 207 | PSMA1 |  |  | 1149 | 0.108 | 0.7124 | Yes |
| 208 | TGIF1 |  |  | 1150 | 0.108 | 0.7138 | Yes |
| 209 | CCDC59 |  |  | 1165 | 0.107 | 0.7137 | Yes |
| 210 | RPL9 |  |  | 1166 | 0.107 | 0.7151 | Yes |
| 211 | PPP4R2 |  |  | 1228 | 0.103 | 0.7097 | No |
| 212 | COPS8 |  |  | 1241 | 0.102 | 0.7098 | No |
| 213 | RPL24 |  |  | 1252 | 0.102 | 0.7100 | No |
| 214 | RPL13A |  |  | 1279 | 0.100 | 0.7085 | No |
| 215 | SLIRP |  |  | 1284 | 0.099 | 0.7094 | No |
| 216 | RPL37 |  |  | 1324 | 0.097 | 0.7063 | No |
| 217 | RPL23 |  |  | 1339 | 0.096 | 0.7060 | No |
| 218 | RPS21 |  |  | 1349 | 0.095 | 0.7063 | No |
| 219 | WTAP |  |  | 1378 | 0.093 | 0.7045 | No |
| 220 | CNTLN |  |  | 1386 | 0.093 | 0.7049 | No |
| 221 | HSPH1 |  |  | 1399 | 0.092 | 0.7048 | No |
| 222 | PAPOLA |  |  | 1439 | 0.090 | 0.7017 | No |
| 223 | SVIP |  |  | 1454 | 0.089 | 0.7014 | No |
| 224 | NDUFB1 |  |  | 1456 | 0.089 | 0.7024 | No |
| 225 | SOX9 |  |  | 1466 | 0.088 | 0.7026 | No |
| 226 | NACA |  |  | 1482 | 0.087 | 0.7021 | No |
| 227 | MRPL50 |  |  | 1487 | 0.087 | 0.7029 | No |
| 228 | CCDC18 |  |  | 1491 | 0.087 | 0.7037 | No |
| 229 | RPL18 |  |  | 1526 | 0.085 | 0.7011 | No |
| 230 | G3BP1 |  |  | 1566 | 0.083 | 0.6979 | No |
| 231 | RBL1 |  |  | 1569 | 0.083 | 0.6987 | No |
| 232 | WEE1 |  |  | 1617 | 0.081 | 0.6946 | No |
| 233 | RSL24D1 |  |  | 1626 | 0.080 | 0.6948 | No |
| 234 | PSMD14 |  |  | 1627 | 0.080 | 0.6959 | No |
| 235 | YBX3 |  |  | 1677 | 0.078 | 0.6915 | No |
| 236 | USP39 |  |  | 1701 | 0.077 | 0.6900 | No |
| 237 | RPS28 |  |  | 1743 | 0.075 | 0.6864 | No |
| 238 | ETF1 |  |  | 1749 | 0.075 | 0.6869 | No |
| 239 | NUP155 |  |  | 1798 | 0.073 | 0.6825 | No |
| 240 | TJP1 |  |  | 1811 | 0.072 | 0.6821 | No |
| 241 | CDC25C |  |  | 1820 | 0.072 | 0.6822 | No |
| 242 | SLC25A3 |  |  | 1824 | 0.071 | 0.6828 | No |
| 243 | TRIAP1 |  |  | 1827 | 0.071 | 0.6836 | No |
| 244 | RPL5 |  |  | 1842 | 0.071 | 0.6830 | No |
| 245 | XPO1 |  |  | 1871 | 0.070 | 0.6808 | No |
| 246 | RPF2 |  |  | 1885 | 0.069 | 0.6803 | No |
| 247 | ODC1 |  |  | 1913 | 0.068 | 0.6782 | No |
| 248 | PBX3 |  |  | 1923 | 0.067 | 0.6781 | No |
| 249 | ACAA2 |  |  | 1924 | 0.067 | 0.6790 | No |
| 250 | RPS18 |  |  | 1926 | 0.067 | 0.6798 | No |
| 251 | SERF2 |  |  | 1979 | 0.065 | 0.6749 | No |
| 252 | ZEB1 |  |  | 1995 | 0.065 | 0.6741 | No |
| 253 | PPP1R15B |  |  | 2009 | 0.064 | 0.6735 | No |
| 254 | CASP3 |  |  | 2028 | 0.064 | 0.6723 | No |
| 255 | EIF1B |  |  | 2035 | 0.063 | 0.6725 | No |
| 256 | EIF3J |  |  | 2081 | 0.062 | 0.6684 | No |
| 257 | DPM1 |  |  | 2135 | 0.060 | 0.6633 | No |
| 258 | CBFB |  |  | 2143 | 0.060 | 0.6633 | No |
| 259 | IMMP1L |  |  | 2152 | 0.059 | 0.6632 | No |
| 260 | PEA15 |  |  | 2161 | 0.059 | 0.6631 | No |
| 261 | BNIP3 |  |  | 2196 | 0.058 | 0.6601 | No |
| 262 | KCTD6 |  |  | 2213 | 0.057 | 0.6591 | No |
| 263 | FXR1 |  |  | 2233 | 0.056 | 0.6577 | No |
| 264 | FTH1 |  |  | 2254 | 0.055 | 0.6562 | No |
| 265 | RPS19 |  |  | 2268 | 0.055 | 0.6555 | No |
| 266 | NET1 |  |  | 2298 | 0.054 | 0.6530 | No |
| 267 | ZFP36L1 |  |  | 2320 | 0.053 | 0.6514 | No |
| 268 | GPN1 |  |  | 2347 | 0.052 | 0.6492 | No |
| 269 | CNN3 |  |  | 2349 | 0.052 | 0.6498 | No |
| 270 | SALL1 |  |  | 2372 | 0.051 | 0.6481 | No |
| 271 | POLR2L |  |  | 2435 | 0.050 | 0.6418 | No |
| 272 | PBDC1 |  |  | 2446 | 0.049 | 0.6414 | No |
| 273 | PIN4 |  |  | 2460 | 0.049 | 0.6406 | No |
| 274 | WDR43 |  |  | 2556 | 0.046 | 0.6307 | No |
| 275 | PPM1D |  |  | 2605 | 0.044 | 0.6259 | No |
| 276 | BET1 |  |  | 2655 | 0.043 | 0.6211 | No |
| 277 | FDFT1 |  |  | 2717 | 0.041 | 0.6148 | No |
| 278 | EIF1AD |  |  | 2738 | 0.041 | 0.6132 | No |
| 279 | RYBP |  |  | 2775 | 0.040 | 0.6097 | No |
| 280 | DARS |  |  | 2795 | 0.039 | 0.6081 | No |
| 281 | NEK4 |  |  | 2803 | 0.039 | 0.6078 | No |
| 282 | ADNP |  |  | 2866 | 0.037 | 0.6015 | No |
| 283 | C1orf61 |  |  | 2879 | 0.037 | 0.6006 | No |
| 284 | MYL6 |  |  | 2880 | 0.037 | 0.6011 | No |
| 285 | OSER1 |  |  | 2915 | 0.036 | 0.5978 | No |
| 286 | WDR36 |  |  | 2971 | 0.035 | 0.5922 | No |
| 287 | ZCCHC3 |  |  | 2972 | 0.035 | 0.5926 | No |
| 288 | CAPZA1 |  |  | 2977 | 0.034 | 0.5927 | No |
| 289 | GLI3 |  |  | 3012 | 0.034 | 0.5893 | No |
| 290 | GPCPD1 |  |  | 3022 | 0.033 | 0.5888 | No |
| 291 | TAF9 |  |  | 3040 | 0.033 | 0.5873 | No |
| 292 | MDM1 |  |  | 3047 | 0.033 | 0.5871 | No |
| 293 | ZFAND2A |  |  | 3053 | 0.033 | 0.5870 | No |
| 294 | ZFP36L2 |  |  | 3122 | 0.031 | 0.5799 | No |
| 295 | PHLPP1 |  |  | 3141 | 0.031 | 0.5783 | No |
| 296 | SP3 |  |  | 3197 | 0.030 | 0.5726 | No |
| 297 | PAX6 |  |  | 3226 | 0.029 | 0.5698 | No |
| 298 | CCDC25 |  |  | 3298 | 0.028 | 0.5623 | No |
| 299 | RSRC2 |  |  | 3348 | 0.027 | 0.5572 | No |
| 300 | TAF8 |  |  | 3412 | 0.026 | 0.5506 | No |
| 301 | PPIL4 |  |  | 3424 | 0.025 | 0.5497 | No |
| 302 | TP53 |  |  | 3495 | 0.024 | 0.5422 | No |
| 303 | ALDH1B1 |  |  | 3502 | 0.024 | 0.5419 | No |
| 304 | AHSA1 |  |  | 3596 | 0.022 | 0.5319 | No |
| 305 | BTBD1 |  |  | 3597 | 0.022 | 0.5322 | No |
| 306 | HSPB1 |  |  | 3629 | 0.022 | 0.5290 | No |
| 307 | PRDX6 |  |  | 3639 | 0.022 | 0.5283 | No |
| 308 | TLK1 |  |  | 3656 | 0.021 | 0.5268 | No |
| 309 | GLI2 |  |  | 3744 | 0.020 | 0.5174 | No |
| 310 | ABHD3 |  |  | 3775 | 0.019 | 0.5143 | No |
| 311 | THUMPD1 |  |  | 3876 | 0.018 | 0.5034 | No |
| 312 | CBWD2 |  |  | 3881 | 0.018 | 0.5032 | No |
| 313 | CHORDC1 |  |  | 4024 | 0.015 | 0.4877 | No |
| 314 | WBP4 |  |  | 4034 | 0.015 | 0.4869 | No |
| 315 | DEDD |  |  | 4069 | 0.014 | 0.4833 | No |
| 316 | DUSP16 |  |  | 4075 | 0.014 | 0.4829 | No |
| 317 | MIER1 |  |  | 4129 | 0.014 | 0.4772 | No |
| 318 | HMGA1 |  |  | 4135 | 0.013 | 0.4768 | No |
| 319 | YAP1 |  |  | 4184 | 0.012 | 0.4717 | No |
| 320 | NFIL3 |  |  | 4213 | 0.012 | 0.4687 | No |
| 321 | RC3H2 |  |  | 4218 | 0.012 | 0.4684 | No |
| 322 | ZDHHC2 |  |  | 4222 | 0.012 | 0.4682 | No |
| 323 | MSI1 |  |  | 4226 | 0.012 | 0.4681 | No |
| 324 | IFT74 |  |  | 4255 | 0.011 | 0.4651 | No |
| 325 | DYRK1A |  |  | 4284 | 0.011 | 0.4621 | No |
| 326 | SREK1 |  |  | 4426 | 0.008 | 0.4466 | No |
| 327 | TPT1 |  |  | 4568 | 0.006 | 0.4310 | No |
| 328 | ZNF433 |  |  | 4576 | 0.006 | 0.4303 | No |
| 329 | FAM162A |  |  | 4622 | 0.005 | 0.4254 | No |
| 330 | HSP90AB1 |  |  | 4656 | 0.005 | 0.4218 | No |
| 331 | TOPORS |  |  | 4717 | 0.004 | 0.4152 | No |
| 332 | VEGFA |  |  | 4721 | 0.004 | 0.4149 | No |
| 333 | TBL1X |  |  | 4741 | 0.004 | 0.4128 | No |
| 334 | IRF2BP2 |  |  | 4745 | 0.004 | 0.4125 | No |
| 335 | SRP14 |  |  | 4770 | 0.003 | 0.4099 | No |
| 336 | TMEM258 |  |  | 4779 | 0.003 | 0.4091 | No |
| 337 | PEG3 |  |  | 4783 | 0.003 | 0.4088 | No |
| 338 | DHX40 |  |  | 4838 | 0.002 | 0.4028 | No |
| 339 | SAR1A |  |  | 4852 | 0.002 | 0.4014 | No |
| 340 | PKM |  |  | 4880 | 0.001 | 0.3984 | No |
| 341 | ANAPC16 |  |  | 4923 | 0.001 | 0.3937 | No |
| 342 | TYW3 |  |  | 5014 | -0.000 | 0.3837 | No |
| 343 | IER5 |  |  | 5041 | -0.001 | 0.3809 | No |
| 344 | HSPA1B |  |  | 5070 | -0.001 | 0.3778 | No |
| 345 | ZNF134 |  |  | 5168 | -0.003 | 0.3670 | No |
| 346 | DNAJB4 |  |  | 5172 | -0.003 | 0.3667 | No |
| 347 | TCF7L2 |  |  | 5199 | -0.003 | 0.3639 | No |
| 348 | KDM3A |  |  | 5222 | -0.004 | 0.3615 | No |
| 349 | CDC42EP4 |  |  | 5335 | -0.005 | 0.3491 | No |
| 350 | NSUN4 |  |  | 5391 | -0.007 | 0.3431 | No |
| 351 | CMBL |  |  | 5463 | -0.008 | 0.3353 | No |
| 352 | EAF1 |  |  | 5505 | -0.009 | 0.3309 | No |
| 353 | ASNS |  |  | 5539 | -0.009 | 0.3273 | No |
| 354 | RNF139 |  |  | 5551 | -0.009 | 0.3262 | No |
| 355 | FOXN2 |  |  | 5769 | -0.013 | 0.3023 | No |
| 356 | RAP2C |  |  | 5838 | -0.014 | 0.2949 | No |
| 357 | MCAM |  |  | 5927 | -0.015 | 0.2853 | No |
| 358 | REST |  |  | 5962 | -0.016 | 0.2818 | No |
| 359 | EFNB1 |  |  | 5978 | -0.016 | 0.2803 | No |
| 360 | ATF4 |  |  | 5987 | -0.016 | 0.2797 | No |
| 361 | CHMP1B |  |  | 6031 | -0.017 | 0.2751 | No |
| 362 | AHI1 |  |  | 6071 | -0.018 | 0.2710 | No |
| 363 | SCAF11 |  |  | 6095 | -0.018 | 0.2687 | No |
| 364 | HSPA4L |  |  | 6125 | -0.019 | 0.2657 | No |
| 365 | HES1 |  |  | 6191 | -0.020 | 0.2588 | No |
| 366 | NFKBIA |  |  | 6199 | -0.020 | 0.2583 | No |
| 367 | TUBE1 |  |  | 6201 | -0.020 | 0.2584 | No |
| 368 | MTHFD2L |  |  | 6324 | -0.022 | 0.2452 | No |
| 369 | GSK3B |  |  | 6331 | -0.022 | 0.2448 | No |
| 370 | TCF7L1 |  |  | 6384 | -0.023 | 0.2393 | No |
| 371 | DDIT3 |  |  | 6433 | -0.024 | 0.2343 | No |
| 372 | ERRFI1 |  |  | 6445 | -0.024 | 0.2334 | No |
| 373 | TMEM170A |  |  | 6509 | -0.025 | 0.2268 | No |
| 374 | EEF1A1 |  |  | 6690 | -0.029 | 0.2071 | No |
| 375 | DCLRE1C |  |  | 6712 | -0.030 | 0.2052 | No |
| 376 | ATF3 |  |  | 6737 | -0.030 | 0.2030 | No |
| 377 | INTS8 |  |  | 6741 | -0.030 | 0.2030 | No |
| 378 | MDK |  |  | 6783 | -0.031 | 0.1989 | No |
| 379 | PHLDA1 |  |  | 6867 | -0.033 | 0.1901 | No |
| 380 | KIAA1217 |  |  | 6958 | -0.035 | 0.1806 | No |
| 381 | PPP1R15A |  |  | 7004 | -0.037 | 0.1761 | No |
| 382 | ZNF844 |  |  | 7032 | -0.037 | 0.1736 | No |
| 383 | RPS27L |  |  | 7139 | -0.040 | 0.1623 | No |
| 384 | NOTCH3 |  |  | 7196 | -0.042 | 0.1567 | No |
| 385 | TNFRSF19 |  |  | 7226 | -0.043 | 0.1540 | No |
| 386 | RDH10 |  |  | 7270 | -0.044 | 0.1499 | No |
| 387 | ZNF217 |  |  | 7333 | -0.045 | 0.1436 | No |
| 388 | GPBP1L1 |  |  | 7340 | -0.046 | 0.1435 | No |
| 389 | TAF7 |  |  | 7341 | -0.046 | 0.1441 | No |
| 390 | TPM1 |  |  | 7414 | -0.048 | 0.1368 | No |
| 391 | ZNF516 |  |  | 7492 | -0.050 | 0.1289 | No |
| 392 | PDCD4 |  |  | 7568 | -0.052 | 0.1213 | No |
| 393 | CNIH1 |  |  | 7659 | -0.055 | 0.1120 | No |
| 394 | TFAP2C |  |  | 7828 | -0.060 | 0.0941 | No |
| 395 | ARHGEF10 |  |  | 7852 | -0.061 | 0.0924 | No |
| 396 | PTAR1 |  |  | 7908 | -0.063 | 0.0871 | No |
| 397 | TSPAN6 |  |  | 8000 | -0.067 | 0.0779 | No |
| 398 | DNAJC1 |  |  | 8012 | -0.068 | 0.0776 | No |
| 399 | TIPARP |  |  | 8047 | -0.069 | 0.0748 | No |
| 400 | SFRP1 |  |  | 8103 | -0.071 | 0.0696 | No |
| 401 | LIFR |  |  | 8134 | -0.073 | 0.0673 | No |
| 402 | ATP1B1 |  |  | 8199 | -0.077 | 0.0612 | No |
| 403 | RFX4 |  |  | 8211 | -0.078 | 0.0610 | No |
| 404 | CREB5 |  |  | 8279 | -0.082 | 0.0547 | No |
| 405 | ADAMTS9 |  |  | 8313 | -0.083 | 0.0521 | No |
| 406 | HMGA2 |  |  | 8329 | -0.084 | 0.0516 | No |
| 407 | CREB3L2 |  |  | 8341 | -0.085 | 0.0515 | No |
| 408 | ZFHX4 |  |  | 8390 | -0.088 | 0.0474 | No |
| 409 | CCNL1 |  |  | 8435 | -0.091 | 0.0437 | No |
| 410 | ARRDC3 |  |  | 8461 | -0.093 | 0.0422 | No |
| 411 | RBM39 |  |  | 8469 | -0.093 | 0.0426 | No |
| 412 | MYADM |  |  | 8479 | -0.094 | 0.0429 | No |
| 413 | HEY1 |  |  | 8555 | -0.100 | 0.0359 | No |
| 414 | GPC4 |  |  | 8568 | -0.101 | 0.0360 | No |
| 415 | MAGED2 |  |  | 8572 | -0.101 | 0.0370 | No |
| 416 | SLC25A37 |  |  | 8632 | -0.105 | 0.0318 | No |
| 417 | VIM |  |  | 8691 | -0.110 | 0.0269 | No |
| 418 | IGF2BP2 |  |  | 8696 | -0.111 | 0.0279 | No |
| 419 | FGFR3 |  |  | 8715 | -0.112 | 0.0274 | No |
| 420 | SPCS1 |  |  | 8717 | -0.113 | 0.0288 | No |
| 421 | OFD1 |  |  | 8748 | -0.117 | 0.0271 | No |
| 422 | AGO3 |  |  | 8766 | -0.118 | 0.0268 | No |
| 423 | TRA2A |  |  | 8773 | -0.119 | 0.0277 | No |
| 424 | JAM3 |  |  | 8782 | -0.120 | 0.0284 | No |
| 425 | SRPRB |  |  | 8832 | -0.127 | 0.0247 | No |
| 426 | HDAC9 |  |  | 8833 | -0.127 | 0.0264 | No |
| 427 | PIK3R3 |  |  | 8840 | -0.127 | 0.0274 | No |
| 428 | MAP1B |  |  | 8945 | -0.141 | 0.0177 | No |
| 429 | TTYH1 |  |  | 9029 | -0.154 | 0.0106 | No |
| 430 | PON2 |  |  | 9030 | -0.154 | 0.0127 | No |
| 431 | BOC |  |  | 9110 | -0.168 | 0.0061 | No |
| 432 | SLC5A3 |  |  | 9147 | -0.177 | 0.0045 | No |
| 433 | ATP6V0E1 |  |  | 9173 | -0.182 | 0.0042 | No |
| 434 | FAT1 |  |  | 9207 | -0.193 | 0.0031 | No |
| 435 | MDM4 |  |  | 9211 | -0.197 | 0.0054 | No |
| 436 | FKBP10 |  |  | 9215 | -0.199 | 0.0078 | No |
| 437 | SLC3A2 |  |  | 9268 | -0.220 | 0.0049 | No |
| 438 | PNRC1 |  |  | 9294 | -0.229 | 0.0052 | No |
| 439 | SEMA5A |  |  | 9315 | -0.243 | 0.0063 | No |
| 440 | HSP90B1 |  |  | 9334 | -0.257 | 0.0077 | No |
| 441 | ITGB8 |  |  | 9362 | -0.294 | 0.0087 | No |
Table: GSEA details [plain text format]

  

Fig 2: RICHARDS\_NOWAKOWSKI\_SCIENCE\_2017\_RG\_EARLY\_UPREG: Random ES distribution      
 Gene set null distribution of ES for **RICHARDS\_NOWAKOWSKI\_SCIENCE\_2017\_RG\_EARLY\_UPREG**
